# Sex differences in patients with heart failure and mildly reduced left ventricular ejection fraction

**DOI:** 10.1038/s41598-023-33733-8

**Published:** 2023-04-26

**Authors:** Zhican Liu, Yunlong Zhu, Sihao Chen, Mingxin Wu, Haobo Huang, Ke Peng, Lingling Zhang, Wenjiao Zhao, Xin Peng, Na Li, Hui Zhang, Yuying Zhou, Yiqun Peng, Jie Fan, Jianping Zeng

**Affiliations:** 1 Graduate Collaborative Training Base of Xiangtan Central Hospital, Hengyang Medical School, University of South China, Hengyang, Hunan 421001 China; 2 Department of Cardiology, Xiangtan Central Hospital, Xiangtan, 411100 China

**Keywords:** Cardiology, Medical research

## Abstract

Clinical studies on heart failure with mildly reduced left ventricular ejection fraction (HFmrEF) have gradually increased. However, studies on the prognostic differences between men and women among patients with HFmrEF are few, and no evidence on sex differences in such patients exists. Therefore, we retrospectively assessed the data of patients with HFmrEF using propensity score-matched analysis (PSMA). A total of 1691 patients with HFmrEF were enrolled in the Outcome of Discharged HFmrEF Patients study (OUDI-HF study), which included 1095 men and 596 women. After propensity score matching, we compared the difference in cardiovascular (CV) events (cardiovascular death or heart failure readmission) and all-cause mortality at 90 days and 1 year after discharge between men and women using Kaplan–Meier analysis and Cox regression. After PSMA, men with HFmrEF were 2.2 times more likely to die at 90 days than women with HFmrEF [hazard ratio (HR) 1.88; 95% confidence interval (95% CI) 1.03–3.46; P = 0.041]. However, there was no difference in the 90-day CV events (HR 0.96; 95% CI 0.75–1.22; P = 0.718). Similarly, there was no difference in all-cause mortality (HR 1.16; 95% CI 0.81–1.65; P = 0.417) and CV events (HR 0.98; 95% CI 0.83–1.16; P = 0.817) between men and women after 1 year. Among the patients with HFmrEF, men had a higher 90-day risk of all-cause mortality than women after hospital discharge, and this risk disappeared after 1 year.

Clinical Trial Registration: URL: http://www.clinicaltrials.gov. Unique identifier: NCT05240118 (*ESC Heart Failure*. (2022). doi: 10.1002/ehf2.14044).

## Introduction

Cardiovascular disease is the most common cause of death in men and women worldwide^1^. Heart failure (HF) is a pandemic that has placed tremendous stress on patients, caregivers, and healthcare systems^2,3^. Based on the left ventricular ejection fraction (LVEF), heart failure can be categorized into heart failure with reduced ejection fraction (HFrEF) and heart failure with preserved ejection fraction (HFpEF). The range between these two fractions has been termed as “HF with mid-range ejection fraction (EF),” or “HF with mildly reduced EF” referring an LVEF of 41–49%^4–6^.

Studies have shown that men are more likely to have HFrEF, whereas women are more likely to have HFpEF^7–12^. However, only few studies exist on sex differences in patients with HFmrEF^13^. Most HF studies worldwide were conducted on men^14^, and information collected about men with HF cannot be assumed to apply equally to women. Therefore, we conducted a retrospective study to compare the differences in outcome events between men and women with HFmrEF.

## Methods

### Study population and data source

The study protocol was approved by the Ethics Committee of Xiangtan Central Hospital (Xiangtan, China, No. 20211036) and conformed to the principles outlined in the Declaration of Helsinki^15^. The Ethics Committee Review Board of Xiangtan Central Hospital waived the need for written informed consent. Consent was obtained from all patients or their guardians during follow-up. This study was based on the Outcome of Discharged HFmrEF Patients study (OUDI-HF study; a retrospective study). The OUDI-HF study included 1691 patients with HFmrEF who were admitted to our hospital from 1 January 2015 to 31 August 2020. The inclusion criteria were HF with an LVEF of 41% to 49% and a New York Heart Association HF score of II to IV.The exclusion criteria were malignancies or other non-cardiac diseases with expected survival of less than 1 year.

### Outcomes

Demographic and procedural data were collected from patients' hospital charts or databases. All study participants were followed up on 31 August 2021. A panel of seven experienced physicians reviewed suspected CV events by examining the information obtained from hospital records and follow-ups, including clinical telephone interviews and community visits. The primary outcome of interest was all-cause death after discharge, and the secondary outcome was the composite of CV death and HF readmissions (CV events). *Cardiovascular death* is death from any cardiovascular mechanism: death from acute myocardial infarction, sudden cardiac death, death from heart failure, death from stroke, death from cardiovascular surgery, death from cardiovascular hemorrhage, and death from other cardiovascular causes. All-cause mortality is death from all causes, including cardiovascular death. Coronary heart disease was defined by coronary angiography evidenced > 50% stenosis of the left main stem, > 70% stenosis in a major coronary vessel, or 30% to 70% stenosis with fractional flow reserve ≤ 0.8. Myocardial infarction was defined by clinical history of ischemic type chest pain lasting for more than 20 min; changes in serial ECG tracings; rise and fall of serum cardiac biomarkers such as creatine kinase-MB fraction and troponin. PCI (percutaneous coronary intervention) referred to minimally invasive procedures used to open clogged coronary arteries.

### Statistical analysis

Continuous variables are expressed as the mean ± standard deviation. The propensity score matching analysis was performed using a multivariate logistic regression model based on the following factors: age, body mass index, current smoker, hypertension, hyperlipidemia, diabetes, coronary heart disease, atrial fibrillation, previous stroke, chronic obstructive pulmonary disease, renal insufficiency, myocardial infarction, history of percutaneous coronary intervention, and New York heart function classification. Pairs of patients, men or women, were derived within a quarter of the standard deviation of the estimated propensity using 1:1 greedy nearest-neighbor matching. This strategy provided 530 matching pairs per group. A COX risk regression model was added to verify the reliability of the statistical results after propensity score matching.

Clinical characteristics between the groups were compared using *t-*tests for continuous measures and chi-squared tests for categorical variables. Non-parametric tests for continuous, not normally distributed variables. The Kaplan–Meier method was used to estimate the incidence of cumulative events. A Cox proportional hazards model was constructed to assess the hazard ratio for each event between the two groups. After propensity score matching, the balance of measured variables between groups was analyzed using paired *t*-tests for continuous measures and McNemar’s test for categorical variables. After propensity score matching, differences in cumulative event rates were analyzed using the stratified Cox procedure.

P-values were obtained using the Kruskal–Wallis rank-sum test for continuous variables and Fisher’s exact test for count variables. Results were considered significant when the *P*-value was less than 0.05. All analyses were performed using R (http://www.R-project.org) and EmpowerStats software (http://www.empowerstats.com, X&Y solutions, Inc. Boston MA).

### Ethics approval and consent to participate

The study protocol was approved by the Ethics Committee of Xiangtan Central Hospital (Xiangtan, China, No. 20211036) and conformed to the principles outlined in the Declaration of Helsinki. The need for informed consent was waived by the ethics committee Review Board of Xiangtan Central Hospital, because of the retrospective nature of the study.

## Results

**Figure 1:**
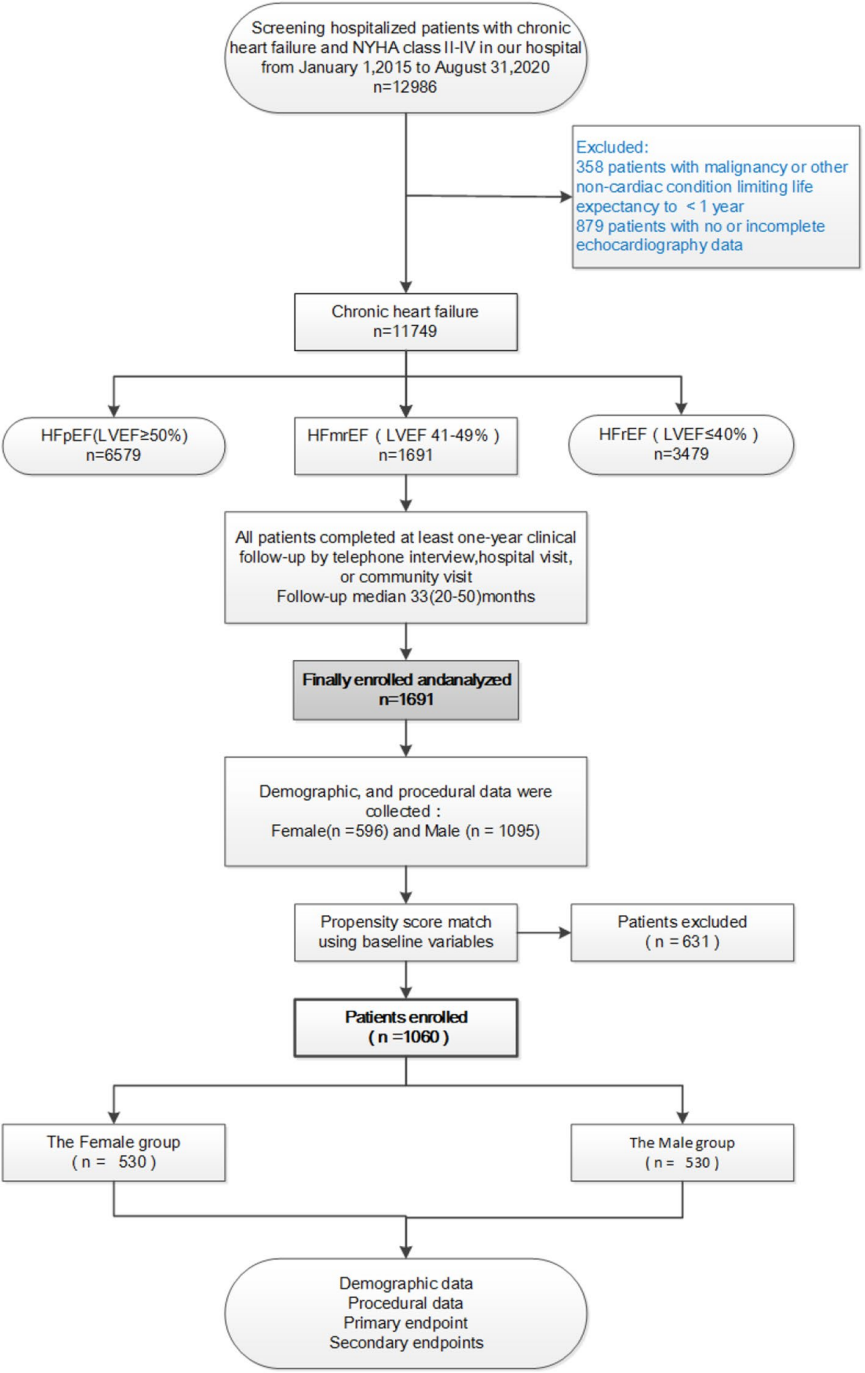
Flow diagram for participant screening, eligibility and analysis.

Among the 1691 patients with HFmrEF, including 1095 men and 596 women, 530 matched pairs were obtained after propensity score matching analysis (Fig. 1). Table 1 shows patient profiles before and after propensity score matching. Before propensity score matching, men had higher rates of current smoking (P < 0.001), coronary heart disease (P = 0.024), chronic obstructive pulmonary disease (P < 0.001), myocardial infarction (P < 0.001), and percutaneous coronary intervention (P < 0.001) ratio than women. Differences in age (P < 0.001) and New York heart function class (P < 0.001) were also noted. Body mass index (P = 0.644), hypertension (P = 0.055), hyperlipidemia (P = 0.087), diabetes (P = 0.203), atrial fibrillation (P = 0.531), previous stroke (P = 0.637), and renal insufficiency (P = 0.596) were similar between the two groups. After propensity score matching, there were no differences in other variables between the two groups and the P-values were both > 0.05, indicating that there was no difference between the two groups and that they were comparable.

**Table 1:** Baseline characteristics before and after propensity-score matching.

Characteristic	Before propensity-score matching			After propensity-score matching		
	Female (N = 596)	Male (N = 1095)	P-value	Female (N = 530)	Male (N = 530)	P-value
Age (year)			< 0.001			0.975
≤ 60 years old	120 (20.13)	295 (26.94)		117 (22.1)	114 (21.5)	
61–74 years old	230 (38.59)	434 (39.63)		215 (40.6)	216 (40.8)	
≥ 75 years old	246 (41.28)	366 (33.42)		198 (37.4)	200 (37.7)	
BMI (kg/m^2^)	25.04 ± 4.30	25.14 ± 4.03	0.644	25.01 ± 4.27	25.06 ± 3.86	0.820
Current smoker (%)	48 (8.05)	496 (45.30)	< 0.001	48 (9.1)	45 (8.5)	0.838
Hypertension (%)	427 (71.64)	735 (67.12)	0.055	378 (71.3)	391 (73.8)	0.409
Hyperlipidemia (%)	137 (22.99)	213 (19.45)	0.087	121 (22.8)	102 (19.2)	0.175
Diabetes mellitus (%)	207 (34.73)	347 (31.69)	0.203	187 (35.3)	186 (35.1)	1
Coronary heart disease (%)	448 (75.17)	875 (79.91)	0.024	401 (75.7)	409 (77.2)	0.613
Atrial fibrillation (%)	109 (18.29)	187 (17.08)	0.531	95 (17.9)	108 (20.4)	0.349
Previous stroke (%)	76 (12.75)	131 (11.96)	0.637	66 (12.5)	72 (13.6)	0.648
COPD (%)	33 (5.54)	176 (16.07)	< 0.001	33 (6.2)	39 (7.4)	0.542
Renal insufficiency (%)	139 (23.32)	268 (24.47)	0.596	131 (24.7)	138 (26)	0.672
NYHA functional class [n (%)]			0.001			0.479
II	226 (37.92)	493 (45.02)		216 (40.8)	217 (40.9)	
III	219 (36.74)	399 (36.44)		191 (36)	205 (38.7)	
IV	151 (25.34)	203 (18.54)		123 (23.2)	108 (20.4)	
Myocardial infarction (%)	265 (44.46)	608 (55.53)	< 0.001	251 (47.4)	256 (48.3)	0.806
PCI (%)	158 (26.51)	407 (37.17)	< 0.001	154 (29.1)	163 (30.8)	0.592

Values are mean ± SD or %.

*BMI* body mass index, *COPD* chronic obstructive pulmonary disease, *NYHA* New York Heart Association, *PCI* percutaneous coronary intervention, *CV event* cardiovascular event (cardiovascular death or heart failure readmission).

Table 2 supplements other baseline information not involved in propensity matching scores. The table shows no difference between male and female HFmrEF patients at baseline in Systolic blood pressure, Beta-blocker, angiotensin receptor blocker, angiotensin receptor neprilysin inhibitor, left ventricular ejection fraction, Left atrial size and interventricular septal depth. However, there are still differences in other indicators. However, most of the baselines became either no difference or a reduced difference after propensity matching scores.

**Table 2:** Baseline characteristics not participating in PSMA matching.

	Before propensity-score matching			After propensity-score matching		
	Female (N = 596)	Male (N = 1095)	P-value	Female (N = 530)	Male (N = 530)	P-value
Age, years	69.6 ± 11.9	67.4 ± 12.5	< 0.001	68.8 ± 12.0	68.8 ± 12.7	0.982
Systolic blood pressure, mmHg	137.3 ± 26.0	136.0 ± 25.8	0.293	137.4 ± 25.5	136.6 ± 26.1	0.61
Heart rate, bpm	85.5 ± 20.5	83.2 ± 19.4	0.019	85.6 ± 20.2	83.1 ± 19.4	0.041
Current drinker, N (%)	8 (1.3%)	139 (12.7%)	< 0.001	8 (1.5%)	17 (3.2%)	0.105
NT-proBNP, pg/ml	8667.7 ± 10,772.1	5943.5 ± 8853.8	< 0.001	8733.3 ± 10,942.6	7347.3 ± 10,048.5	0.046
eGFR, ml/min/1.73 m^2^	64.6 ± 36.4	72.2 ± 34.5	< 0.001	64.6 ± 36.5	67.1 ± 33.9	0.242
Treatment, N (%)						
Beta-blocker	476 (79.9%)	874 (79.8%)	0.981	428 (80.8%)	416 (78.5%)	0.402
ACEi	280 (47.0%)	579 (52.9%)	0.02	257 (48.5%)	254 (47.9%)	0.902
ARB	176 (29.5%)	276 (25.2%)	0.055	151 (28.5%)	145 (27.4%)	0.732
ARNI	25 (4.2%)	54 (4.9%)	0.493	21 (4%)	32 (6%)	0.159
SGLT2i	6 (1.0%)	3 (0.3%)	0.048	5 (0.9%)	0 (0%)	0.073
Lipid-regulating drugs	335 (56.2%)	560 (51.1%)	0.046	437 (82.5%)	427 (80.6%)	0.476
Spironolactone	263 (44.1%)	512 (46.8%)	0.3	229 (43.2%)	254 (47.9%)	0.139
Echocardiography						
LVEF, %	44.3 ± 2.8	44.5 ± 2.7	0.346	44.4 ± 2.8	44.4 ± 2.8	0.696
LAs (mm)	39.3 ± 6.4	39.3 ± 6.1	0.975	39.1 ± 6.4	40.4 ± 6.1	0.001
LVd (mm)	52.5 ± 6.9	54.8 ± 6.8	< 0.001	52.4 ± 6.9	55.6 ± 6.8	< 0.001
IVSd (mm)	9.9 ± 1.5	10.1 ± 1.6	0.002	9.9 ± 1.6	10.3 ± 1.6	< 0.001
LVPWd (mm)	9.4 ± 1.4	9.6 ± 1.6	0.015	9.4 ± 1.4	9.7 ± 1.7	0.012
RAs (mm)	36.8 ± 6.0	38.1 ± 6.3	< 0.001	36.7 ± 5.9	38.7 ± 6.8	< 0.001
RVd (mm)	20.3 ± 5.0	21.2 ± 5.4	< 0.001	20.3 ± 5.0	21.6 ± 5.9	< 0.001
E/e’	17.0 ± 8.4	15.1 ± 7.2	< 0.001	16.9 ± 8.3	16.1 ± 7.9	0.118
PASP (mmHg)	35.0 ± 16.0	31.6 ± 18.2	< 0.001	34.9 ± 16.0	33.5 ± 20.3	0.185

Values are mean ± SD or %.

*PSMA *propensity score-matched analysis, *NT-proBNP* N-terminal pro-B type natriureti peptide, *eGFR *estimated glomerular filtration rate, *ACEi *angiotensin-converting enzyme inhibitor, *ARB *angiotensin receptor blocker, *ARNI *angiotensin receptor neprilysin inhibitor, *SGLT2i *sodium-dependent glucose transporters 2 inhibitor, *LVEF *left ventricular ejection fraction, *LAs* left atrial size, *LVd* left ventricle dimension, *IVSd* interventricular septal depth, *LVPWd* left ventricular posterior wall decreased, *RAs* right atrial size, *RVd* right ventricle dimension, *E/e*’ ratio between peak early diastolic velocity and early diastolic tissue velocity, *PASP *pulmonary artery systolic pressure.

Table 3 presents the risk of primary and secondary outcomes in patients before and after the propensity score-matched cohort. Before propensity score matching, risk of cardiovascular events was similar between men and women with HFmrEF within 90 days (event rate:22.6% vs. 23.0%, HR 0.98; 95% CI 0.79–1.21; P = 0.836) or 1 year (46.8% vs. 49.7%, HR 0.92; 95% CI 0.80–1.06; P = 0.269). There was no difference between males and females in the incidence of all-cause mortality at 90 days (4.2% vs. 3.4%, HR 1.25; 95% CI 0.74–2.11; P = 0.406) or 1 year (11.5% vs.11.6%, HR 0.99; 95% CI 0.74–1.33; P = 0.970).

**Table 3:** Risk of primary and secondary outcomes before and after propensity-score-matched cohort.

Outcome	Before propensity-score matching				After propensity-score matching			
	No. of event	Event rate (%)	Hazard ratio (95% CI)	P value	No. of event	Event rate (%)	Hazard ratio (95% CI)	P value
90 day all-cause death								
Female	16	3.0	Reference		20	3.4	Reference	
Male	30	5.7	1.25 (0.74, 2.11)	0.406	46	4.2	1.88 (1.03, 3.46)	0.041
1 year all-cause death								
Female	57	10.8	Reference		69	11.6	Reference	
Male	65	12.3	0.99 (0.74, 1.33)	0.970	126	11.5	1.16 (0.81, 1.65)	0.417
90 day CV events								
Female	128	24.2	Reference		137	23.0	Reference	
Male	123	23.2	0.98 (0.79, 1.21)	0.836	248	22.6	0.96 (0.75, 1.22)	0.718
1 year CV events								
Female	264	49.8	Reference		296	49.7	Reference	
Male	261	49.2	0.92 (0.80, 1.06)	0.269	513	46.8	0.98 (0.83, 1.16)	0.817

The propensity-score-matched cohort included 530 patients in the male group and 530 patients in the female group.

After propensity score matching, men with HFmrEF were 1.88 times more likely to die at 90 days than women with HFmrEF (mortality: 5.7% vs. 3.0% for men and women, hazard ratio (HR) 1.88; 95% confidence interval (95% CI) 1.03–3.46; P = 0.041). The difference in all-cause mortality between men and women was not significant after 1 year of follow-up (12.3% vs. 10.8%, HR 1.16; 95% CI 0.81–1.65; P = 0.417) (Table 3). No difference was noted in 90-day or 1-year cardiovascular event rates between men and women (Table 3). The difference in the presence of all-cause mortality at 90 days between male and female HFmrEF persisted after COX multiple regression analysis and adjustment for confounders (HR 1.84; 95% CI 1.02–3.34; P = 0.045) (Table 4).

**Table 4:** Results of a multivariate Cox proportional hazards model for the effect of gender on outcome events in patients with HFmrEF.

Outcome	Non-adjusted hazard ratio (95% CI)	*P*-value	Adjust I hazard ratio (95% CI)	*P*-value	Adjust II hazard ratio (95% CI)	*P*-value
Ninety day all-cause death						
Female	Reference					
Male	1.25 (0.74, 2.11)	0.406	1.38 (0.82, 2.34)	0.228	1.84 (1.02, 3.34)	0.045
One year all-cause death						
Female	Reference					
Male	0.99 (0.74, 1.33)	0.970	1.09 (0.81, 1.46)	0.586	1.33 (0.94, 1.89)	0.104
Ninety day CV events						
Female	Reference					
Male	0.98 (0.79, 1.21)	0.836	1.02 (0.83, 1.26)	0.861	1.09 (0.86, 1.39)	0.467
One year CV events						
Female	Reference					
Male	0.92 (0.80, 1.06)	0.269	0.95 (0.83, 1.10)	0.524	1.00 (0.85, 1.19)	0.966

Adjust I model adjust for: age.

Adjust II model adjust for: age; body mass index; current smoker; current drinker; hypertension; hyperlipidemia; diabetes mellitus; coronary heart disease; atrial fibrillation; previous stroke; chronic obstructive pulmonary disease; renal insufficiency; New York Heart Association; eGFR; NT-proBNP; heart rate; systolic blood pressure; myocardial infarction; percutaneous coronary intervention; estimated glomerular filtration rate.

**Figure 2:**
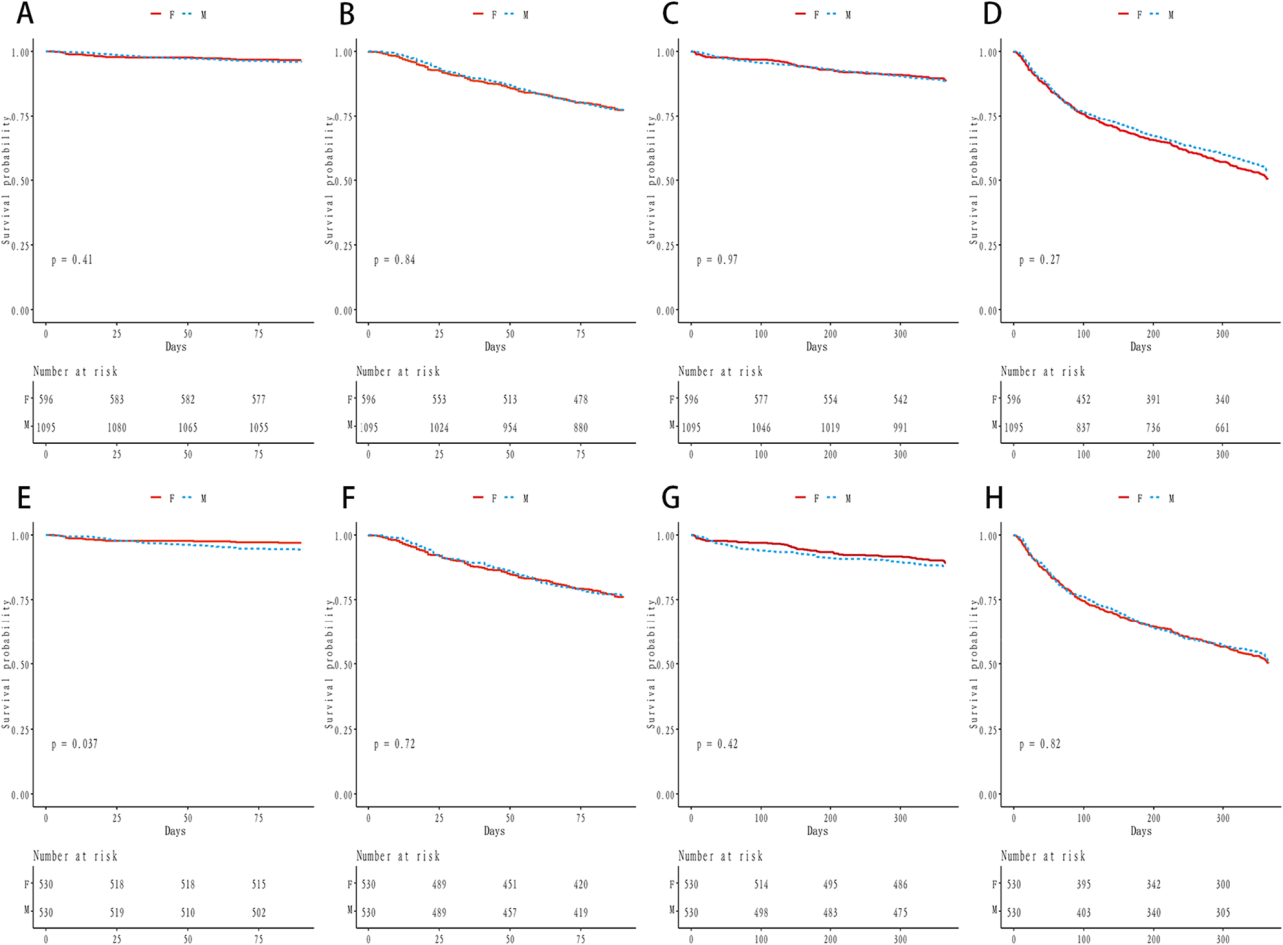
Kaplan–Meier curves of outcomes before and after PSM matching. (**A**) The cumulative 90-day all-cause mortality (before PSM matching). (**B**) The cumulative 90-day CV events (before PSM matching). (**C**) The cumulative 1 year all-cause death (before PSM matching). (**D**) The cumulative 1 year CV events (before PSM matching). (**E**) The cumulative 90-day all-cause mortality (after PSM matching). (**F**) The cumulative 90-day CV events (after PSM matching). (**G**) The cumulative 1 year all-cause death (after PSM matching). (**H**) The cumulative 1 year CV events (after PSM matching).

In Kaplan–Meier survival curves before propensity score matching was performed, there was no difference between male and female HFmrEF on 90 days all-cause mortality (P = 0.41) and one-year all-cause mortality (P = 0.97) (Fig. 2A, C). Figure 2B, D shows no difference in the incidence of cardiovascular events between men and women within 90 days (P = 0.84) and one-year (P = 0.27).

In Kaplan–Meier survival curves after propensity score matching, the cumulative 90-day all-cause mortality was higher in men than in women (P = 0.037) (Fig. 2E). The all-cause mortality difference between men and women gradually disappeared after a year (P = 0.42)(Fig. 2G). There was no difference in cardiovascular events within 90 days and one year between men and women, shown in Fig. 2F (P = 0.72) and Fig. 2H (P = 0.82).

Table 5 shows the subgroup analysis of male and female HFmrEF patients on 90-day all-cause mortality. Compared with women, this group of patients with a BMI < 30, no history of atrial fibrillation, no history of COPD and no history of PCI had a higher all-cause mortality rate at 90 days in the male HFmrEF (P < 0.05).

**Table 5:** Comparison of 90 day all cause deaths between men and women.

	All		Ninety day all-cause death		Hazard ratio (95% CI)	*P*-value
	Female (N = 530) (%)	Male (N = 530) (%)	Female (N = 16) (%)	Male (N = 30) (%)		
Age						
≤ 60 years old	117 (22.1)	114 (21.5)	1 (6.2)	2 (6.7)	2.1 (0.2, 22.7)	0.555
61–74 years old	215 (40.6)	216 (40.8)	3 (18.8)	9 (30.0)	3.0 (0.8, 11.1)	0.100
≥ 75 years old	198 (37.4)	200 (37.7)	12 (75.0)	19 (63.3)	1.6 (0.8, 3.3)	0.214
Body mass index						
< 30	398 (75.1)	422 (79.6)	8 (50.0)	24 (80.0)	2.9 (1.3, 6.4)	**0.010**
≥ 30	132 (24.9)	108 (20.4)	8 (50.0)	6 (20.0)	0.9 (0.3, 2.6)	0.857
Current smoker						
No	482 (90.9)	485 (91.5)	16 (100.0)	25 (83.3)	1.6 (0.8, 2.9)	0.168
Yes	48 (9.1)	45 (8.5)	0 (0.0)	5 (16.7)	Inf. (0.0, Inf)	0.999
Hypertension						
No	152 (28.7)	139 (26.2)	0 (0.0)	8 (26.7)	Inf. (0.0, Inf)	0.998
Yes	378 (71.3)	391 (73.8)	16 (100.0)	22 (73.3)	1.3 (0.7, 2.5)	0.389
Hyperlipidemia						
No	409 (77.2)	428 (80.8)	14 (87.5)	26 (86.7)	1.8 (0.9, 3.4)	0.080
Yes	121 (22.8)	102 (19.2)	2 (12.5)	4 (13.3)	2.4 (0.4, 12.9)	0.320
Diabetes mellitus						
No	343 (64.7)	344 (64.9)	9 (56.2)	19 (63.3)	2.1 (1.0, 4.7)	0.062
Yes	187 (35.3)	186 (35.1)	7 (43.8)	11 (36.7)	1.6 (0.6, 4.1)	0.348
Coronary heart disease						
No	129 (24.3)	121 (22.8)	3 (18.8)	5 (16.7)	1.8 (0.4, 7.4)	0.436
Yes	401 (75.7)	409 (77.2)	13 (81.2)	25 (83.3)	1.9 (1.0, 3.7)	0.060
Atrial fibrillation						
No	435 (82.1)	422 (79.6)	11 (68.8)	22 (73.3)	2.1 (1.0, 4.3)	**0.049**
Yes	95 (17.9)	108 (20.4)	5 (31.2)	8 (26.7)	1.4 (0.5, 4.3)	0.545
Previous stroke						
No	464 (87.5)	458 (86.4)	14 (87.5)	24 (80.0)	1.7 (0.9, 3.4)	0.098
Yes	66 (12.5)	72 (13.6)	2 (12.5)	6 (20.0)	2.8 (0.6, 13.6)	0.215
Chronic obstructive pulmonary disease						
No	497 (93.8)	491 (92.6)	14 (87.5)	29 (96.7)	2.1 (1.1, 4.0)	**0.021**
Yes	33 (6.2)	39 (7.4)	2 (12.5)	1 (3.3)	0.4 (0.0, 4.5)	0.467
Renal insufficiency						
No	399 (75.3)	392 (74.0)	10 (62.5)	14 (46.7)	1.4 (0.6, 3.2)	0.387
Yes	131 (24.7)	138 (26.0)	6 (37.5)	16 (53.3)	2.5 (1.0, 6.5)	0.051
New York Heart Association functional class						
II	216 (40.8)	217 (40.9)	3 (18.8)	10 (33.3)	3.4 (0.9, 12.2)	0.066
III	191 (36.0)	205 (38.7)	6 (37.5)	10 (33.3)	1.6 (0.6, 4.3)	0.391
IV	123 (23.2)	108 (20.4)	7 (43.8)	10 (33.3)	1.6 (0.6, 4.3)	0.324
Myocardial infarction						
No	279 (52.6)	274 (51.7)	9 (56.2)	18 (60.0)	2.1 (0.9, 4.6)	0.078
Yes	251 (47.4)	256 (48.3)	7 (43.8)	12 (40.0)	1.7 (0.7, 4.3)	0.273
Percutaneous coronary intervention						
No	376 (70.9)	367 (69.2)	13 (81.2)	25 (83.3)	2.0 (1.0, 3.9)	**0.046**
Yes	154 (29.1)	163 (30.8)	3 (18.8)	5 (16.7)	1.6 (0.4, 6.7)	0.525

Bold represent significant values (p < 0.05).

*CI* confidence interval.

## Discussion

The main finding of this study was that men with HFmrEF had a higher risk of all-cause mortality 90 days after discharge than women with HFmrEF, particularly in the interval of 20–80 days after discharge.

Previous studies showed that women with HF without further classification of LVEF were treated in smaller proportions but still had better outcomes than men ^13,16–20^. The Framingham Heart Study (FHS), which was conducted from 1990 to 1999, showed that women with HF without further classification of LVEF had better survival rates than men, with age-adjusted 5-year mortality rates of 45% and 59%, respectively ^21^. In HFrEF, the Prospective Comparison of Angiotensin Receptor Neprilysin Inhibitor with Angiotensin Converting Enzyme Inhibitor to Determine Impact on Global Mortality and Morbidity in Heart Failure and Aliskiren Trial to Minimize Outcomes in Patients with Heart failure trials, which included a total of 12,058 men and 3,357 women, showed that all-cause mortality was lower in women than in men (adjusted HR: 0.68; 95% CI: 0.62–0.74; P < 0.001) ^19^. In the Irbesartan in Heart Failure with Preserved Ejection Fraction trial, which included 2491 women, showed a 20% reduction in the risk of all-cause mortality or hospitalization in women even after adjusting for age differences and other baseline characteristics^22^. Moreover, similar conclusions were drawn in studies such as the Candesartan in Heart failure: Assessment of Reduction in Mortality and Morbidity^17^, Beta-Blocker Evaluation of Survival Trial^23^, and Prevention of REnal and Vascular ENdstage Disease^12^. Studies such as FHS and ours demonstrated differences in outcomes between men and women with HF. However, Studies such as FHS failed to explain the sex differences in outcomes of HFmrEF, whereas we attempted to study the sex differences in HFmrEF. Indeed, some studies have reported different outcomes than those reported in the abovementioned studies. In the Olmsted County Heart Failure Events Study from 2000 to 2010, age-adjusted all-cause mortality was similar for women and men^24^. The Atherosclerosis Risk in Communities study (2005–2014) showed that age-adjusted 28-day and 1-year mortality was equally high among men and women hospitalized for acute HF^25^.

Pharmacokinetics is also different caused by differences in body composition between men and women^26^, which result in higher rates of adverse events in women using drugs according to HF guidelines^27,28^. Although the treatment of HF varies in some regions between men and women^8,29^, many disadvantageous factors still exist for women with HF. For instance, women with HF have a poorer quality of life and continue to receive suboptimal treatment^19^, even less access to a cardiologist^30,31^, and lower use of left ventricular assist devices^32,33^. Male heart failure patients were independently associated with cardiac death but not with a composite endpoint or all-cause mortality^34^. Although the annual mortality rate is higher in men, more women than men die of heart failure each year, and the clinical presentation of heart failure differs between men and women^35^.Therefore, more research is required to assess the different treatments available for HF between men and women. Moreover, it needs to stratify and recruit more women in HF trials.

Proportion of cardiovascular or noncardiovascular deaths were presented in the Table S1. Results showed that after PSM both cardiovascular and noncardiovascular death rates are both borderline higher in men than in women (P = 0.107 and 0.101, respectively), thus the higher all-cause deaths in men might be the jointly effects of borderline higher cardiovascular and noncardiovascular deaths in men.Our analysis showed that both cardiovascular and noncardiovascular deaths rates are borderline higher in men than in women within 90 days post discharge, the joint effects of cardiovascular and noncardiovascular deaths might explain the significantly higher all-cause death within 90 days post discharge in our cohort. It is to note that this study is a single center retrospective analysis, which suffers the nature of selection bias in this study setting, future prospective multi-center clinical trials are needed to validate our finding. The study also failed to explain why women had better outcomes than men in heart failure. Women have better heart failure outcomes, presumably related to different risk factors and hormone levels in men and women with heart failure^31^. For example, the leading cause of heart failure in male patients is ischaemic heart disease, while in female patients, the leading cause of heart failure is atrial fibrillation^11^. This is consistent with our baseline data, where male patients had less atrial fibrillation and more coronary heart disease than female patients (Table 1). In fact, our study showed that female patients had both borderline lower risk of cardiovascular and noncardiovascular death at 90 days post discharge. More research is needed to advance this area to improve the treatment of both female and male heart failure patients.

### Limitations

This study has several limitations. First, this was a retrospective study to minimize bias in patient selection, but unobserved confounders remained. Second, our study exclusively recruited patients from China from an isolated population at a local heart center, thereby lacking diversity to justify the uniformity of the findings. Lastly, we could not include all variables that differed between men and women in the analysis of propensity matching scores. This difference may affect the outcome to some extent.

## Conclusions

After propensity score matching, men with HFmrEF had a higher risk of all-cause mortality 90 days after discharge than women with HFmrEF. This risk disappeared after one year. This higher risk of all-cause mortality in men is mainly seen in those with less severe underlying disease. So, attention should be paid to short-term survival after discharge in men with HFmrEF, regardless of their severity. There were no differences in the 90-day and 1-year cardiovascular events between men and women with HFmrEF. Further research is warranted to understand the complex sex-related risk differences among patients with HF. A better understanding of sex-specific risk factors may help develop strategies to improve outcomes in this critical disease.

## Supplementary Information

Supplementary Information.

Supplementary Table S1.

## Data Availability

The datasets generated and analyzed during the current study are not publicly available due the database owner is reluctant to make them public but are available from the corresponding author upon reasonable request.

## Abbreviations

HFmrEF: Heart failure with mid-range or mildly reduced ejection fraction
PSMA: Propensity score-matched analysis
CV: Cardiovascular
HR: Hazard ratio
CI: Confidence interval
HF: Heart failure
EF: Ejection fraction
LVEF: Left ventricular ejection fraction
HFrEF: Heart failure reduced ejection fraction
HFpEF: Heart failure preserved ejection fraction
BMI: Body mass index
COPD: Chronic obstructive pulmonary disease
NYHA: New York Heart Association
PCI: Percutaneous coronary intervention
FHS: The Framingham Heart Study
NT-proBNP: Terminal pro-B type natriureti peptide
eGFR: Estimated glomerular filtration rate
ACEi: Angiotensin-converting enzyme inhibitor
ARB: Angiotensin receptor blocker
ARNI: Angiotensin receptor neprilysin inhibitor
SGLT2i: Sodium-dependent glucose transporters 2 inhibitor
LAs: Left atrial size
LVd: Left ventricle dimension
IVSd: Interventricular septal depth
LVPWd: Left ventricular posterior wall decreased
RAs: Right atrial size
RVd: Right ventricle dimension
e/e: Ratio between peak early diastolic velocity and early diastolic tissue velocity
PASP: Pulmonary artery systolic pressure
