# Supplementary material for: Sex differences in patients with heart failure and mildly reduced left ventricular ejection fraction
Source: Sci Rep. 2023 Apr 26;13:6832. doi: 10.1038/s41598-023-33733-8 (PMC10133291; doi:10.1038/s41598-023-33733-8)
Supplement: Supplementary file 2 — Supplementary Table S1. [file 41598_2023_33733_MOESM2_ESM.docx]

| **Table S1. 90-day incidence of all-cause and cardiovascular mortality before and after Propensity-Score Matching.** | | | | | | |
| --- | --- | --- | --- | --- | --- | --- |
|  | Before Propensity-Score Matching | | | After Propensity-Score Matching | | |
|  | Female | Male | P-value | Female | Male | P-value |
| ninety day all-cause deaths | 20 (3.4%) | 46 (4.2%) | 0.391 | 16 (3.0%) | 30 (5.7%) | 0.035 |
| ninety day cardiovascular deaths | 19 (3.2%) | 38 (3.5%) | 0.759 | 15 (2.8%) | 25 (4.7%) | 0.107 |
| ninety day noncardiovascular deaths | 1(0.2%) | 8(0.7%) | 0.129 | 1(0.2%) | 5(1.0%) | 0.101 |
